# Non-Selective β-Blocker Use and Portal Vein Thrombosis in Cirrhosis: An Updated Systematic Review and Meta-Analysis

**DOI:** 10.3390/biomedicines14092034

**Published:** 2026-09-10

**Authors:** Sanchuan Lai, Wei Wei, Tingting Su

**Affiliations:** 1 Department of Gastroenterology, The Second Affiliated Hospital, Zhejiang University School of Medicine, Hangzhou 310009, China; lsanchuan@zju.edu.cn (S.L.); wwze@zju.edu.cn (W.W.); 2 Department of Gastroenterology, The First Affiliated Hospital, Zhejiang University School of Medicine, Hangzhou 310003, China

**Keywords:** non-selective β-blocker, portal vein thrombosis, cirrhosis, meta-analysis

## Abstract

**Background/Objectives**: The association between non-selective β-blocker (NSBB) use and portal vein thrombosis (PVT) in patients with cirrhosis remains controversial. Prior meta-analyses had notable methodological limitations and may have overestimated this relationship. This updated systematic review and meta-analysis aimed to comprehensively evaluate the association between NSBB therapy and PVT risk in cirrhotic populations. **Methods**: We systematically searched PubMed, Web of Science, the Cochrane Library, and EMBASE up to 24 January 2026. Observational clinical studies comparing PVT incidence between cirrhotic patients receiving NSBBs and those not receiving NSBB treatment were included. The Newcastle–Ottawa Scale (NOS) was used for methodological quality assessment. Pooled odds ratios (ORs) and hazard ratios (HRs) with 95% confidence intervals (CIs) were calculated separately using random-effects models. Subgroup, sensitivity, and publication bias analyses were performed to explore heterogeneity and verify result robustness. **Results**: Eighteen observational studies enrolling 8104 cirrhotic patients were finally included. For binary PVT endpoints from 12 studies, NSBB use was significantly associated with elevated PVT risk (pooled OR = 1.89, 95% CI 1.29–2.78), with moderate-to-high between-study heterogeneity (I^2^ = 67.8%). For longitudinal incident PVT evaluated by HRs in 6 cohort studies, the overall pooled association did not reach statistical significance (HR = 1.61, 95% CI 0.99–2.63). Subgroup analyses demonstrated that the effect size was further attenuated after multivariable adjustment. **Conclusions**: Observational evidence reveals a correlation between NSBB exposure and higher PVT prevalence in cirrhotic patients. However, adjusted longitudinal data fail to confirm that NSBB therapy independently increases the risk of de novo PVT. Large-scale, well-adjusted prospective cohort studies are needed to verify the relationship between NSBB administration and incident PVT.

## 1. Introduction

Liver cirrhosis, a progressive fibrotic liver disease affecting approximately 2.2 million adults in the United States alone, is characterized by the disruption of liver architecture and the development of portal hypertension, a key driver of life-threatening complications [1,2]. Portal vein thrombosis (PVT) refers to the formation of non-malignant thrombus in the main trunk of the portal vein and/or its intrahepatic or extrahepatic branches, which can lead to partial or complete obstruction of portal blood flow [3,4]. As a critical complication of cirrhosis, PVT has an annual incidence ranging from 4.6% to 26% in cirrhotic patients [4,5], and its prevalence varies with disease stage, diagnostic methods, and patient populations [6,7]. PVT not only exacerbates portal hypertension but also increases the risk of variceal rebleeding, hepatic decompensation, and mortality [8]. In severe cases, it may contraindicate liver transplantation or impair post-transplant survival [4,9]. The pathogenesis of PVT in cirrhosis is multifactorial, involving the three components of Virchow’s triad: reduced portal blood flow velocity, hypercoagulable state and endothelial dysfunction [3,6].

Non-selective β-blockers (NSBBs) are a class of medications that non-specifically block both β1 and β2 adrenergic receptors. Commonly used agents include propranolol, carvedilol, and nadolol, which exert dual pharmacological effects: blocking β1 receptors reduces cardiac output, while blocking β2 receptors induces splanchnic vasoconstriction [10]. As cornerstone therapies for the primary and secondary prevention of variceal bleeding in cirrhotic patients with portal hypertension [1,10], NSBBs reduce portal vein inflow and portal pressure through the above mechanisms. However, their potential impact on PVT development has sparked intense clinical debate in recent years. The core controversy lies in their dual hemodynamic effects: while NSBBs reduce bleeding risk, they may also decrease portal vein blood flow velocity, creating a milieu conducive to thrombus formation [4,11]. Low shear stress upregulates endothelial expression of prothrombotic factors (tissue factor, von Willebrand factor) while downregulating anticoagulant mediators (thrombomodulin, endothelial protein C receptor) [12,13]. Additionally, NSBB-induced reduction in hepatic perfusion may indirectly impair hepatic synthetic function and perturb coagulation balance, an effect more pronounced in patients with higher Child–Pugh grades [14,15,16].

Given the widespread clinical use of NSBBs and the severe consequences of PVT, clarifying their relationship is crucial for optimizing the management of cirrhotic patients with portal hypertension. Previous systematic reviews and meta-analyses have focused primarily on NSBBs’ efficacy in preventing variceal bleeding, with few specifically addressing their impact on PVT risk [17,18]. Existing clinical studies have inconsistent conclusions [3,4,11], and potential modifying factors remain unclear. Although a meta-analysis investigating the relationship between NSBBs and PVT was published in 2019 [19], 3 out of the 9 included studies were conference abstracts rather than full-text articles. Furthermore, only 6 full-text studies were included in that analysis, among which 3 received a Newcastle–Ottawa Scale (NOS) score of merely 6, indicating suboptimal methodological quality. The limited number and suboptimal quality of included studies may have introduced potential bias, thereby undermining the robustness and generalizability of their conclusions. In addition, a large number of clinical studies on the association between NSBBs and PVT have been published from 2019 to the present, providing more abundant and updated research data for this research field. Therefore, this study conducts a meta-analysis of all existing relevant full-text articles to systematically evaluate the association between NSBB use and PVT development in cirrhotic patients.

## 2. Materials and Methods

### 2.1. Study Design and Registration

This meta-analysis was designed as a systematic evaluation of clinical studies, and the study protocol was prospectively registered in the International Prospective Register of Systematic Reviews (PROSPERO), and the registration number is CRD420261327839. The reporting of this study strictly followed the Preferred Reporting Items for Systematic Reviews and Meta-Analyses (PRISMA) statement.

### 2.2. Publication Selection Criteria

No language restrictions were applied to the study selection process. All studies meeting the following eligibility criteria were included: those that compared the risk of portal vein thrombosis (PVT) between cirrhotic patients who received non-selective β-blockers (NSBBs) and those who did not receive NSBB treatment. Exclusion criteria were as follows: (1) duplicate publications; (2) systematic reviews and meta-analyses; (3) correspondence articles; (4) case reports and case series with fewer than 10 participants; (5) studies lacking adequate supporting details; (6) cross-sectional studies (given their inherent inability to establish the temporal sequence between NSBB exposure and PVT development, which precludes valid causal inference and may introduce significant bias); (7) overlapping cohorts.

### 2.3. Literature Search Strategy

We conducted comprehensive searches of four electronic databases—PubMed, Web of Science, the Cochrane Library, and EMBASE—to retrieve all publications documenting the incidence of portal vein thrombosis (PVT) in cirrhotic patients with and without non-selective β-blockers (NSBBs) exposure. The search strategy incorporated the key terms liver cirrhosis or cirrhotic in combination with portal vein thrombosis and NSBBs. Detailed search strategies can be found in Supplementary File S2. The final database search was conducted on 24 January 2026. Two independent investigators (S.L. and T.S.) screened the titles and abstracts of all identified studies to exclude those that failed to meet the predefined eligibility criteria. Any discrepancies arising during this screening process were resolved by consultation with a third reviewer (W.W.).

### 2.4. Data Extraction

Standardized data extraction forms were developed, and two investigators independently extracted data from eligible studies using these forms (double entry in STATA 17). The extracted core information includes the first author, publication year, country, study type, follow-up time, total sample size, and NOS score. Hazard ratios (HRs) with 95% CI (Confidence Interval) derived from Cox regression models were extracted for cohort studies. If a cohort study did not report HRs, corresponding odds ratios (ORs) for binary PVT status were extracted instead. For case–control studies, odds ratios (ORs) were extracted as the standard effect measure.

For both HR and OR estimates, multivariable-adjusted effect values (adjusted HR/adjusted OR) were prioritized for extraction to reduce confounding by indication and baseline imbalances in disease severity. Crude unadjusted effect estimates (unadjusted HR/unadjusted OR) were extracted only when multivariable-adjusted results were not available in the original publication. As for studies with missing key data, the corresponding research authors will be contacted via email to supplement the data; if the data cannot be obtained after contact, the studies will be excluded.

### 2.5. Quality Assessment of Included Studies

Two researchers independently evaluate the methodological quality and bias risk of the included observational studies, and the inconsistent results are resolved by discussion or third-party arbitration. We adopt the Newcastle–Ottawa Scale (NOS), which includes 8 items in 3 dimensions (selection of study subjects, comparability, exposure/outcome assessment), with a full score of 9 points; studies with NOS scores ≥ 7 points are defined as high-quality studies, and those with <7 points are low-quality studies.

### 2.6. Data Synthesis and Statistical Analysis

All statistical analyses were conducted using Stata software version 17.0 (StataCorp LP, College Station, TX, USA), with a two-sided significance level set at α = 0.05. Heterogeneity across studies was assessed via Cochrane’s Q test and I^2^ statistic. Heterogeneity was considered to be statistically significant if *p* ≤ 0.1 and/or I^2^ ≥ 50%. Effect sizes were pooled separately according to the statistical metrics reported in original studies. Specifically, studies providing odds ratios (ORs) with 95% confidence intervals (CIs) and hazard ratios (HRs) for time-dependent incident PVT outcomes were synthesized independently. For each study, fully multivariable-adjusted effect sizes were preferentially extracted when available; otherwise, crude unadjusted estimates were adopted. Overall pooled estimates combining both adjusted and unadjusted studies are presented for completeness and comparability with prior meta-analyses, but adjusted subgroup estimates represent the most reliable evidence for assessing the independent effect of NSBBs on PVT, given the strong risk of confounding by indication inherent in observational studies. Given the substantial between-study heterogeneity observed across datasets, a random-effects model was applied for all pooled analyses.

Prespecified subgroup analyses were performed differentially for the two datasets. For the OR dataset, subgroup stratification was conducted according to adjustment status (adjusted vs. unadjusted effect sizes), study design, PVT outcome type (incident PVT vs. non-incident PVT), geographic region and methodological quality (NOS score ≥ 7 vs. <7) to explore potential sources of heterogeneity. For the HR dataset, only subgroup analysis based on adjustment status (adjusted vs. unadjusted effect sizes) was performed, as included studies were homogeneous in study type, PVT outcome type, geographic region and methodological quality. Leave-one-out sensitivity analysis was performed for both datasets by sequentially excluding each individual study and re-estimating the pooled effect. Funnel plots and Egger’s test were constructed to assess potential publication bias for the OR dataset comprising 12 studies. In contrast, formal publication bias tests were not conducted for the HR dataset, as the use of fewer than 10 studies yields insufficient power to detect reliable funnel plot asymmetry.

We assessed the certainty of evidence for the primary outcomes using the GRADE (Grading of Recommendations Assessment, Development and Evaluation) framework. Five domains were evaluated for evidence downgrading: risk of bias, inconsistency, indirectness, imprecision, and publication bias. Two independent GRADE evaluations were performed separately for HR-based and OR-based datasets, and full detailed assessment results were summarized in Supplementary Table S4.

## 3. Results

### 3.1. Literature Search and Study Selection

**Figure 1:**
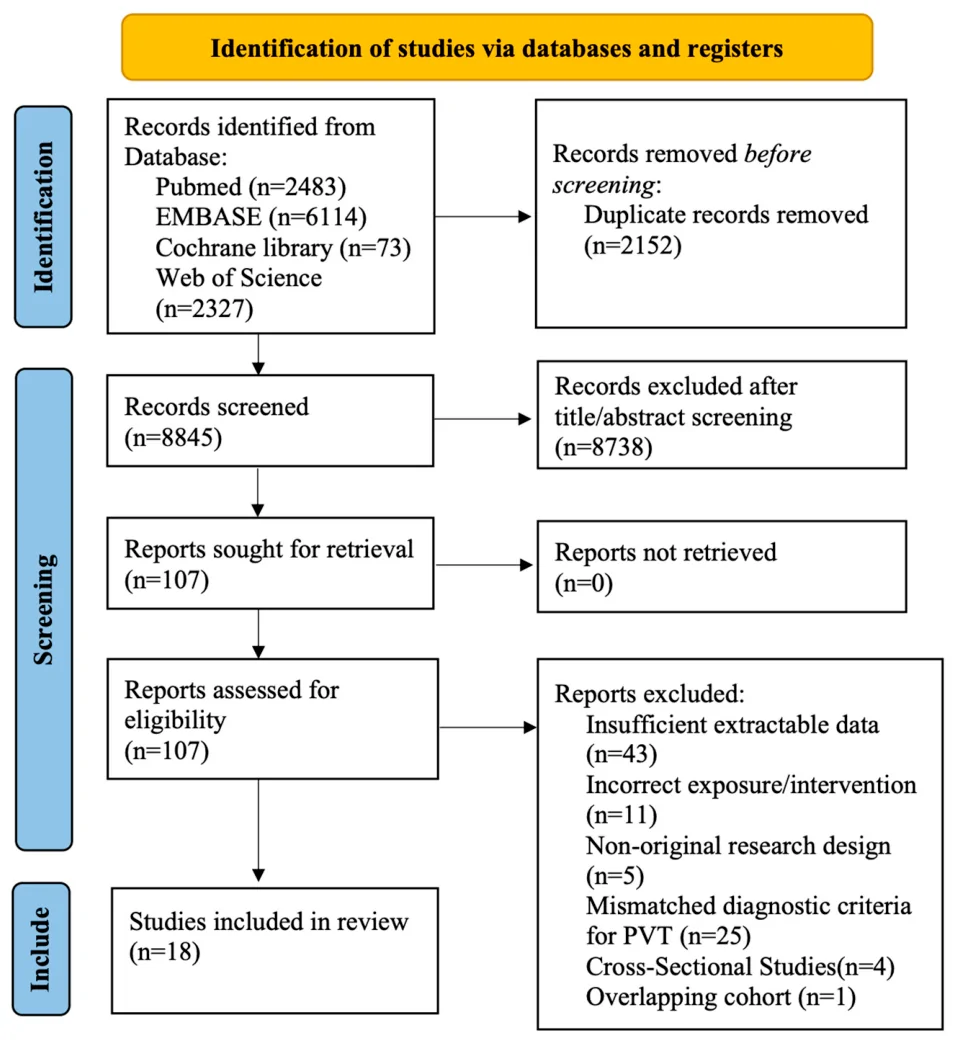
PRISMA flow diagram. Study selection process for the systematic review and meta-analysis. PVT: portal vein thrombosis.

The initial systematic literature search retrieved 10,997 records from pre-specified databases (PubMed, Embase, Cochrane Library and Web of Science). After duplicate removal, 8845 records underwent title and abstract screening, and 8738 irrelevant studies were excluded for non-target population, incorrect exposure/intervention, irrelevant outcomes, or non-original research design. Full-text assessment was performed for 107 potentially eligible studies, among which 84 were excluded for reasons including insufficient extractable data and mismatched diagnostic criteria for liver cirrhosis or portal vein thrombosis (PVT), 4 studies were excluded as cross-sectional studies, and 1 study was excluded for overlapping cohort. Finally, 18 studies were included in the quantitative meta-analysis [3,4,5,6,9,11,20,21,22,23,24,25,26,27,28,29,30,31], enrolling a total of 8104 cirrhotic patients. The study selection process is presented in the PRISMA flow diagram (Figure 1).

### 3.2. Basic Characteristics of Included Studies

Baseline characteristics of the 18 included studies are summarized in Table 1. Of all eligible studies, 6 reported hazard ratios (HRs) and 12 reported odds ratios (ORs) for the association between NSBB use and PVT. For study design, 11 were cohort studies, and 7 were case–control studies. Geographically, 13 studies were conducted in European regions, and 5 were from Asian regions. The NOS scores of included studies ranged from 6 to 9, with 15 studies rated as high-quality (NOS score ≥ 7). The detailed quality assessment results for each study are shown in Tables S2 and S3. All included studies clearly defined the diagnostic criteria for liver cirrhosis and PVT, and the NSBBs evaluated mainly included propranolol and carvedilol. Baseline clinical characteristics, including age, gender, cirrhosis etiology, and Child–Pugh classifications, were generally balanced between the NSBB groups and control groups in most included studies.

### 3.3. Association Between NSBB Use and PVT Development

**Figure 2:**
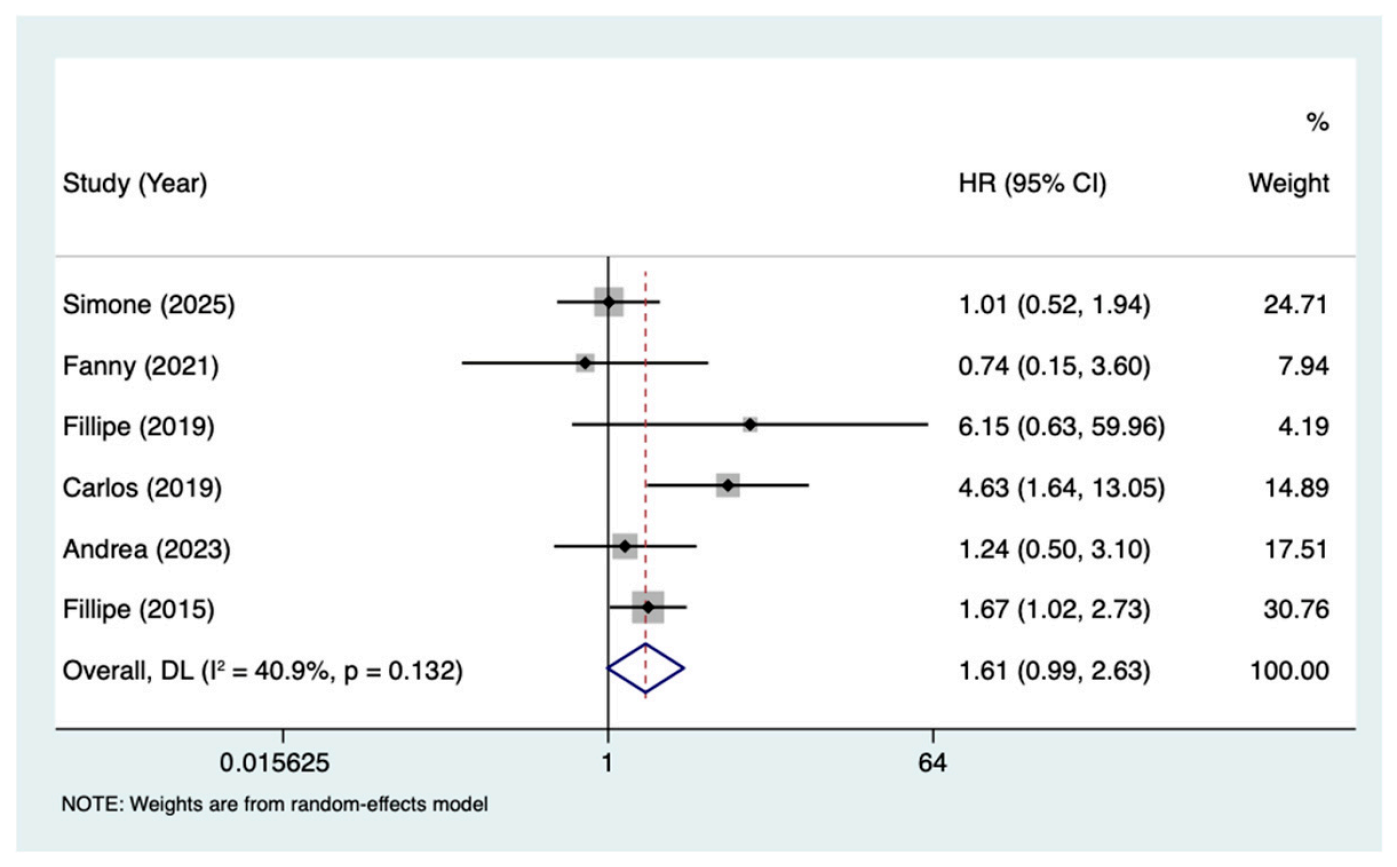
Forest plot of pooled hazard ratios (HRs) for incident portal vein thrombosis (PVT) in cirrhotic patients receiving non-selective β-blockers (NSBBs). Each grey square denotes the HR of an individual study, and the horizontal line represents its 95% CI. The dashed vertical line is the line of no effect (HR = 1). The rhombus indicates the pooled HR and its corresponding 95% CI. CI, confidence interval [3,4,5,20,22,28].

**Figure 3:**
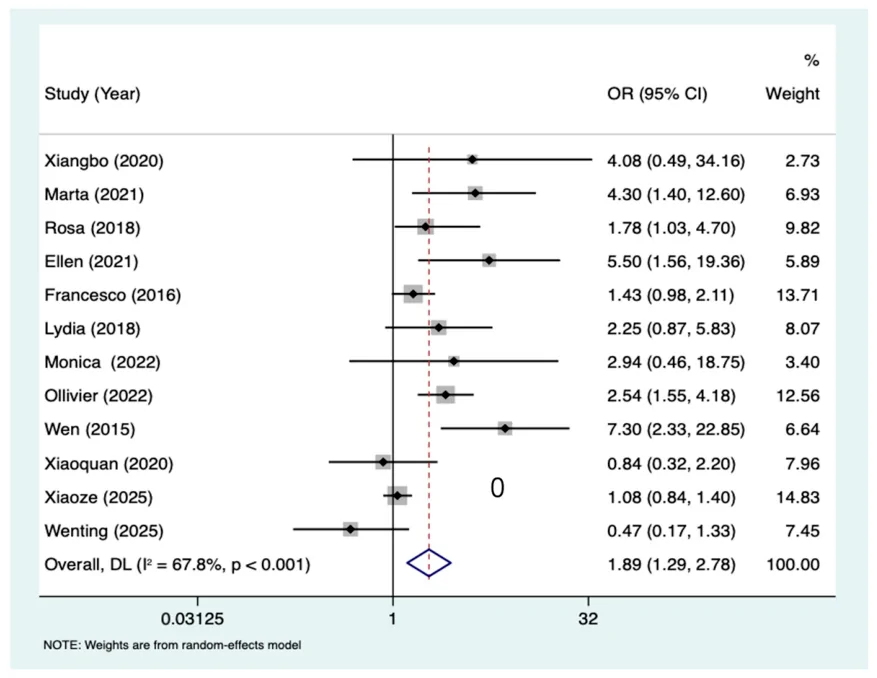
Forest plot of pooled odds ratios (ORs) evaluating the correlation between non-selective β-blocker (NSBB) administration and portal vein thrombosis (PVT) risk in cirrhosis. Each grey square denotes the OR of an individual study, and the horizontal line represents its 95% CI. The dashed vertical line is the line of no effect (OR = 1). The rhombus indicates the pooled OR and its corresponding 95% CI. CI, confidence interval [6,9,11,21,23,24,25,26,27,29,30,31].

The primary outcome was the pooled estimates of PVT development in cirrhotic patients with NSBB exposure versus non-NSBB controls. For the six cohort studies reporting hazard ratios (HRs) for incident PVT during follow-up, moderate heterogeneity existed (I^2^ = 40.9%, *p* = 0.132). The pooled longitudinal estimate indicated a non-significant trend toward higher thrombotic risk among NSBB users (HR = 1.61, 95% CI 0.99, 2.63; Figure 2). For the 12 studies providing odds ratios (ORs) for binary PVT endpoints, substantial heterogeneity was detected (I^2^ = 67.8%, *p* < 0.001). Pooled results revealed a significant positive association between NSBB use and PVT risk (OR = 1.89, 95% CI 1.29, 2.78; Figure 3).

### 3.4. Subgroup Analyses

**Figure 4:**
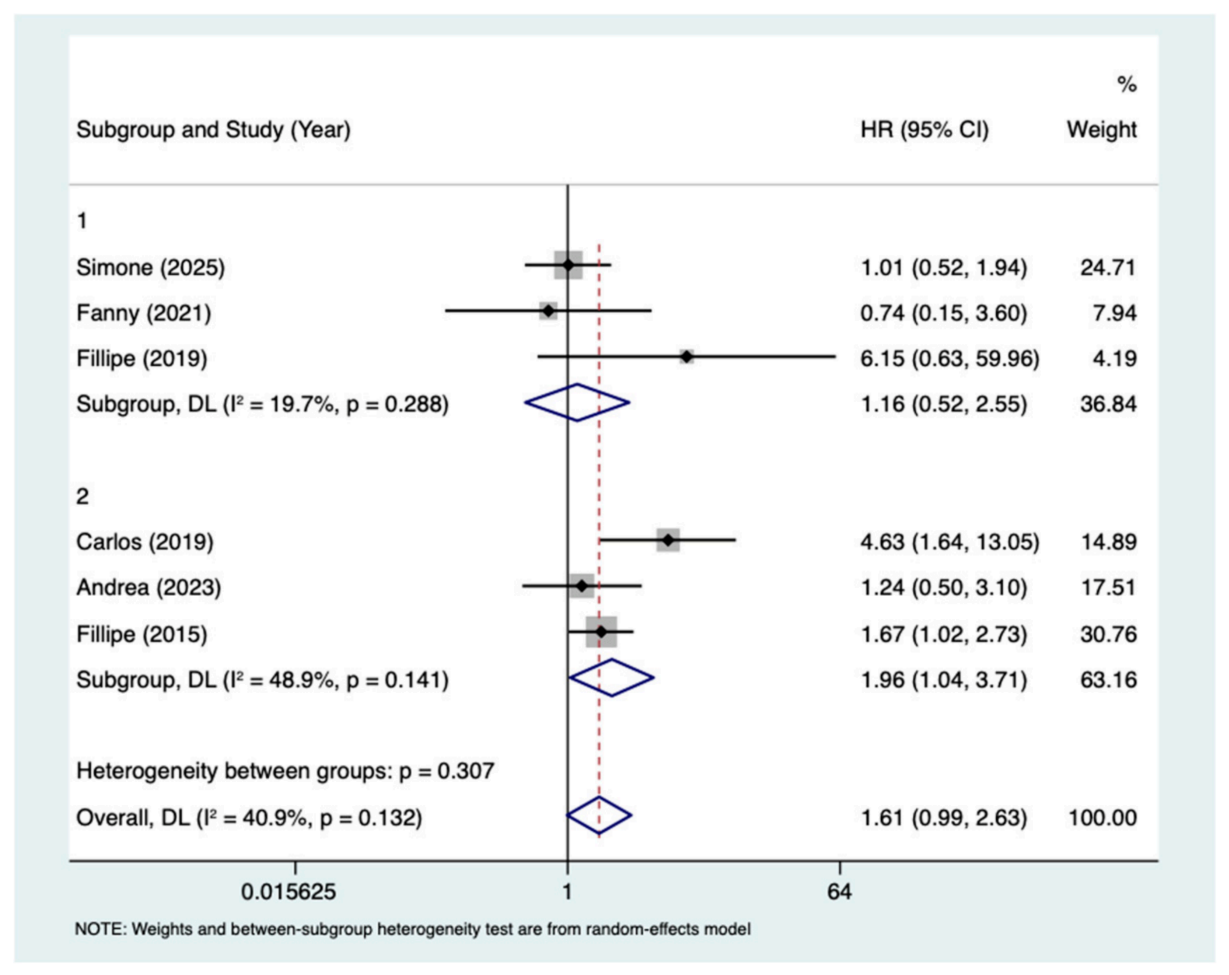
Subgroup forest plot of pooled hazard ratios (HRs) for the association between NSBB exposure and incident PVT in cirrhosis, stratified by adjustment status of effect estimates. Subgroup 1: studies with fully adjusted HRs. Subgroup 2: studies with crude unadjusted HRs. Each grey square denotes the HR of an individual study, and the horizontal line represents its 95% CI. The dashed vertical line is the line of no effect (HR = 1). The rhombus indicates the pooled HR and its corresponding 95% CI. CI: confidence interval [3,4,5,20,22,28].

For the six cohort studies reporting HRs for incident PVT (de novo thrombosis occurring during follow-up), only adjustment status was selected as the stratification factor for subgroup analyses given consistent study design, geographic distribution, and methodological quality across all six longitudinal cohorts. In the adjusted cohort subgroup, NSBB use remained non-significant as regards PVT incidence (HR:1.16, 95% CI (0.52, 2.55), I^2^ = 19.7%, *p* = 0.288), while in the crude cohort subgroup the results indicated otherwise (HR:1.96, 95% CI (1.04, 3.71), I^2^ = 48.9%, *p* = 0.141) (Figure 4). Given the risk of confounding by indication in observational studies of NSBB therapy, adjusted estimates are given primary interpretive weight.

To explore potential sources of heterogeneity in the primary pooled OR estimates, subgroup analyses were performed stratified by adjustment status, study design, PVT outcome type, geographic region, and methodological quality (Table 2). Firstly, we stratified analyses according to whether studies presented multivariable-adjusted or unadjusted effect estimates. Three studies reported fully adjusted ORs, yielding a pooled OR of 2.47 (95% CI 1.36–4.50) with zero between-study heterogeneity (I^2^ = 0.0%, *p* = 0.385). The remaining nine unadjusted studies generated a pooled OR of 1.74 (95% CI 1.12–2.69), accompanied by substantial heterogeneity (I^2^ = 72.5%, *p* < 0.001). Although both subgroups demonstrated a statistically significant positive association between NSBB exposure and PVT risk, heterogeneity was substantially eliminated among studies reporting adjusted estimates (Table 2, Figure S1). Stratifications by study design yielded divergent statistical significance between subgroups. Among five cohort studies reporting ORs, NSBB use was significantly associated with elevated PVT risk (pooled OR = 2.19, 95% CI 1.40–3.42), with low heterogeneity (I^2^ = 36.0%, *p* = 0.182). By contrast, the seven case–control studies failed to reach statistical significance (pooled OR = 1.68, 95% CI 0.94–3.01) and exhibited substantial heterogeneity (I^2^ = 73.0%, *p* = 0.001) (Table 2, Figure S2). We further stratified analyses by PVT outcome definition to explicitly distinguish incident de novo thrombosis from non-incident (prevalent) PVT. Three studies focused on incident PVT, showing a significant pooled association (OR = 2.79, 95% CI 1.80–4.32) with no heterogeneity (I^2^ = 0.0%, *p* = 0.692). The nine studies evaluating non-incident PVT also detected a significant positive association (OR = 1.63, 95% CI 1.07–2.50), alongside moderate-to-high heterogeneity (I^2^ = 66.2%, *p* = 0.003) (Table 2, Figure S3). Subgroup analysis stratified by geographic region revealed inconsistent results across populations. The seven European studies demonstrated a statistically significant higher PVT risk among patients receiving NSBBs (pooled OR = 2.19, 95% CI 1.55–3.09, I^2^ = 29.3%, *p* = 0.204). No significant association was observed in the five Asian studies (pooled OR = 1.41, 95% CI 0.64–3.11), which carried substantial between-study heterogeneity (I^2^ = 73.2%, *p* = 0.005) (Table 2, Figure S4). Finally, stratification by methodological quality based on the NOS score showed significant associations in both subgroups. Three low-quality studies (NOS < 7) reported a pooled OR of 2.27 (95% CI 1.03–4.97, I^2^ = 71.7%, *p* = 0.029), while nine high-quality studies (NOS ≥ 7) yielded a pooled OR of 1.81 (95% CI 1.09–3.00, I^2^ = 69.2%, *p* = 0.001) (Table 2, Figure S5).

### 3.5. Sensitivity Analysis

Leave-one-out sensitivity analyses were conducted separately for six HR-based cohort studies (incident PVT) and twelve OR-based studies to test result robustness.

**Figure 5:**
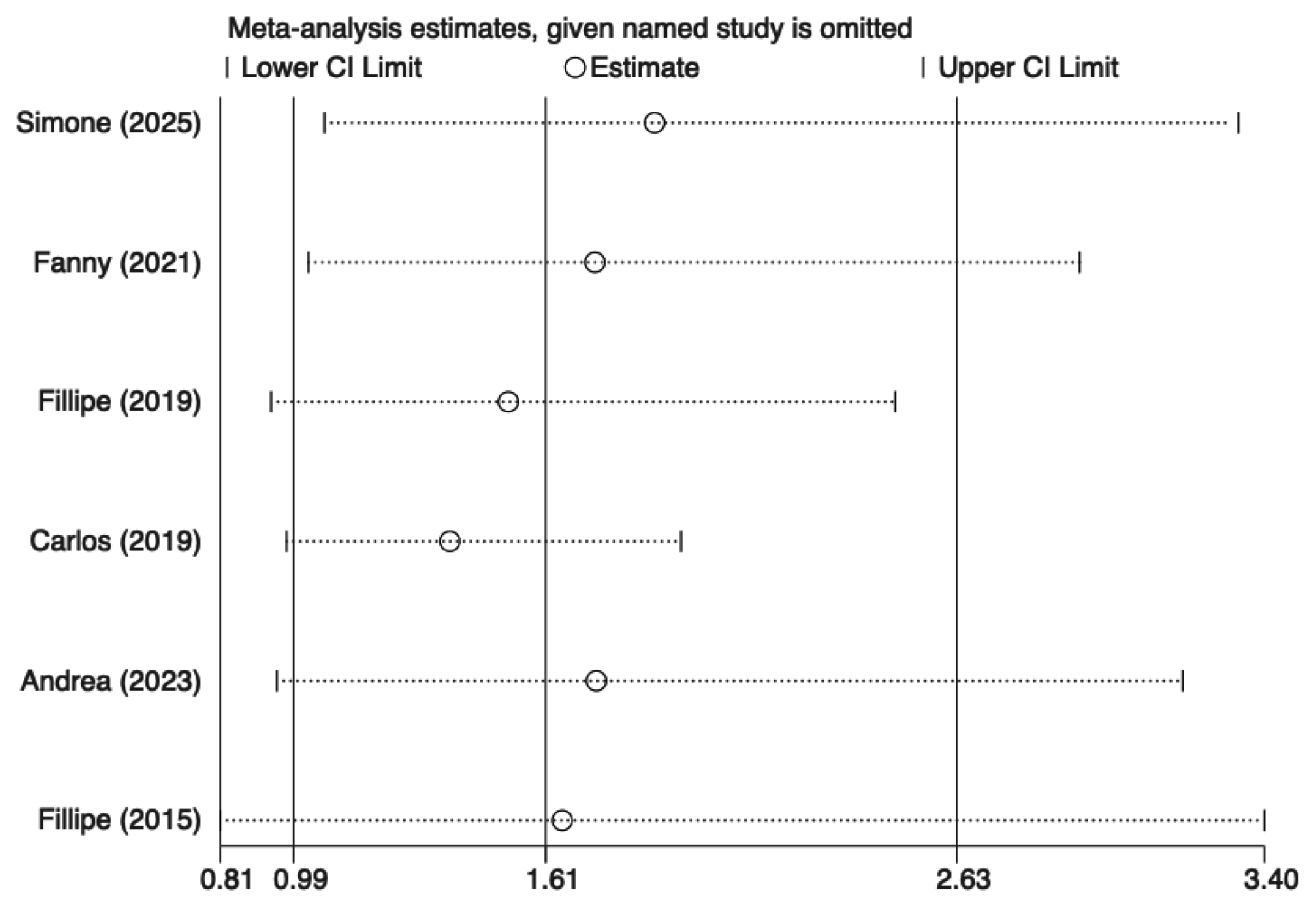
Sensitivity analysis. Leave-one-out analysis for six HR-based cohort studies. CI: confidence interval [3,4,5,20,22,28].

The full cohort pooled HR was 1.61 (95% CI 0.99–2.63), without statistical significance. After omitting individual studies, pooled HRs ranged from 1.38 to 1.88 (Figure 5). Excluding Simone [22] (HR = 1.88, 95% CI 1.07–3.33) or Fanny [3] (HR = 1.74, 95% CI 1.03–2.94) yielded significant positive associations, whereas removing the remaining four studies generated non-significant estimates crossing the null value of 1.

**Figure 6:**
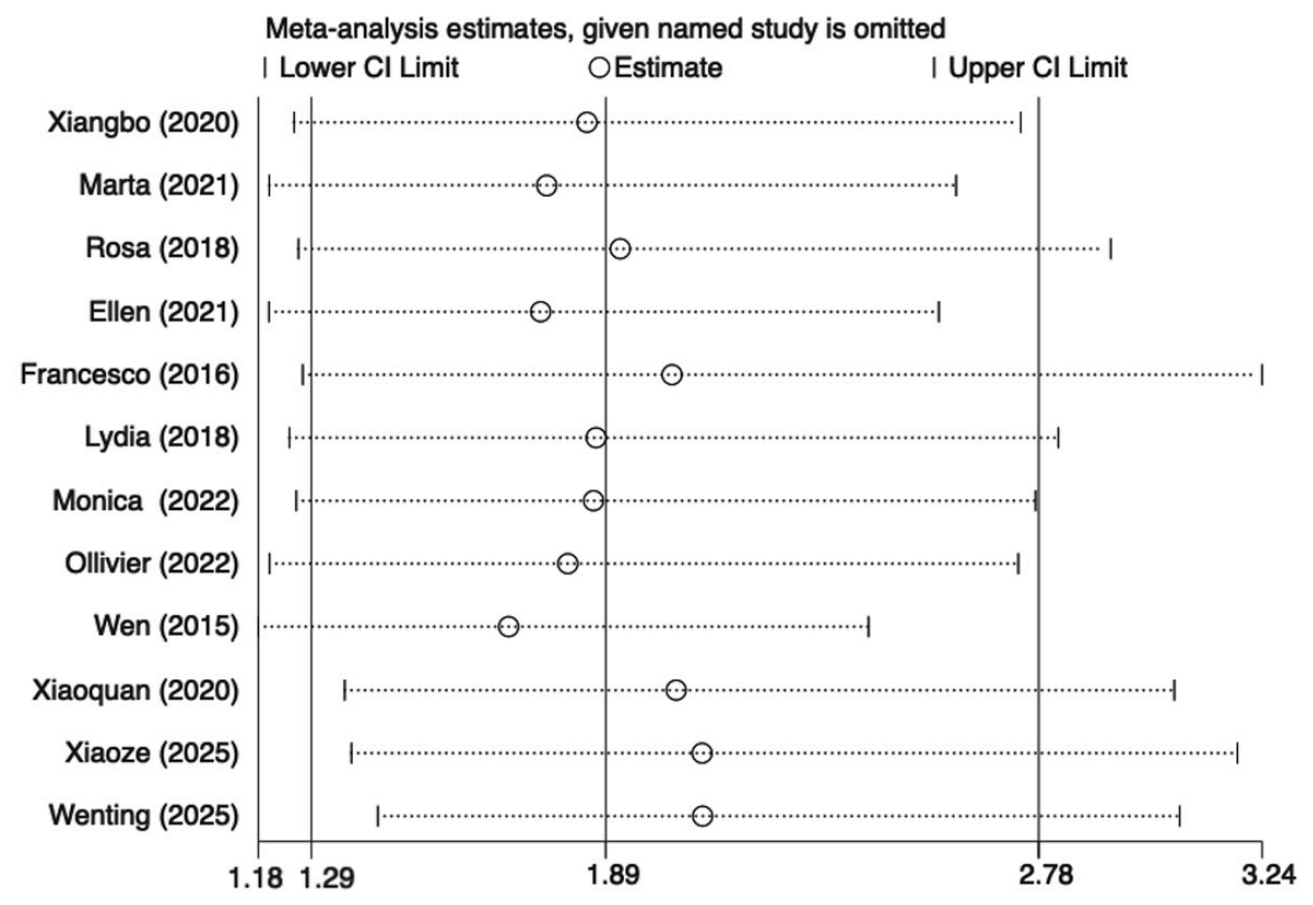
Sensitivity analysis. Leave-one-out analysis for 12 OR-containing studies. CI: confidence interval [6,9,11,21,23,24,25,26,27,29,30,31].

For the 12 OR-containing studies, sequential single-study exclusion induced trivial fluctuations (overall OR = 1.89, 95% CI 1.29–2.78). All recalculated estimates and CIs stayed within the original confidence range (Figure 6).

### 3.6. Publication Bias Assessment

**Figure 7:**
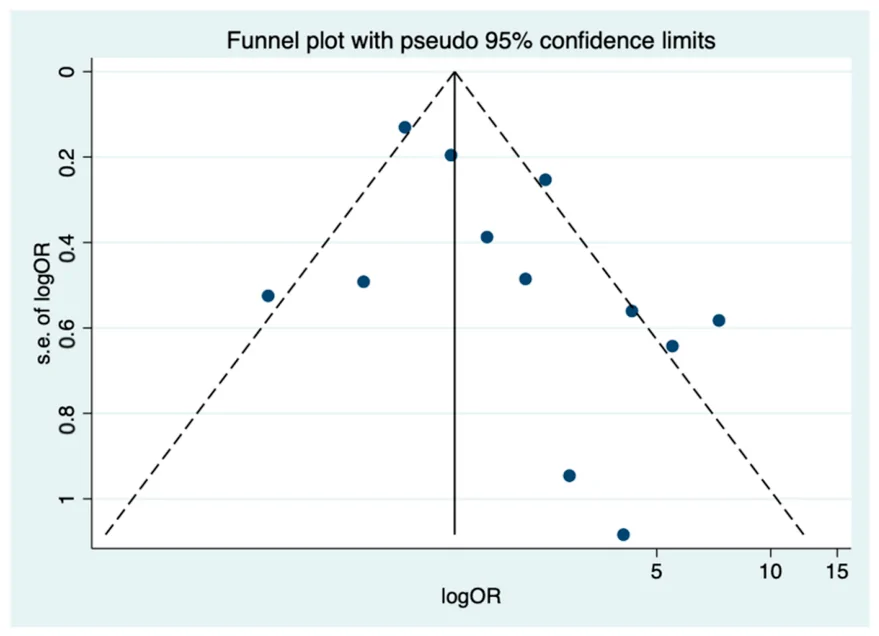
Funnel plot. Solid horizontal line represents the pooled logOR. The dashed lines denote the pseudo 95% confidence limits. Mild asymmetry was observed in the funnel plot.

**Figure 8:**
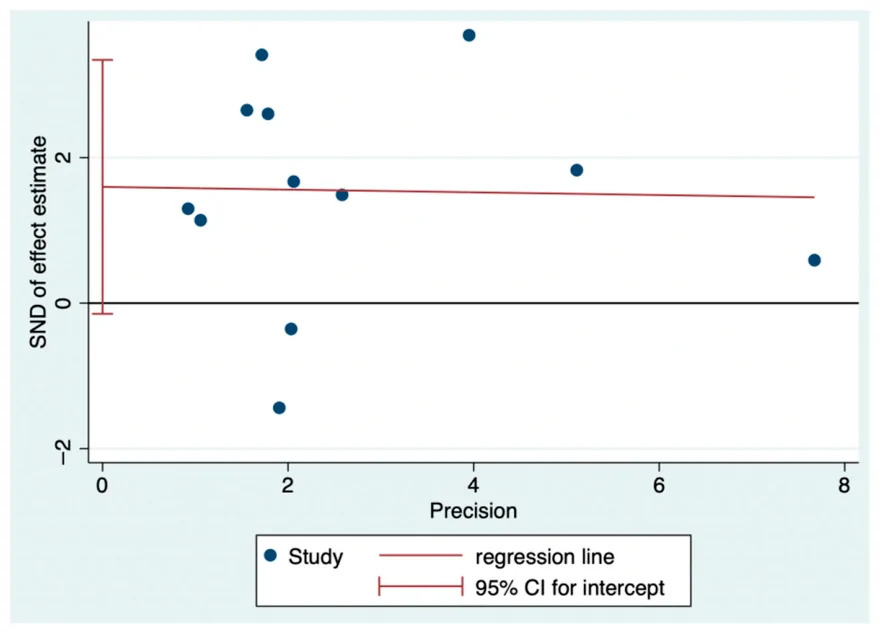
Egger’s test. Egger’s test indicated no significant publication bias.

Publication bias for the 12 studies reporting odds ratios (ORs) was evaluated through visual funnel plot assessment and Egger’s regression test. Mild asymmetry could be visually detected in the funnel plot (Figure 7). Egger’s test of small-study effects generated an intercept of 1.60 with a *p* value of 0.069 (Figure 8), indicating no significant evidence of publication bias was detected across the included OR-based studies.

### 3.7. GRADE Certainty of Evidence

The GRADE framework was used to evaluate evidence certainty (Supplementary Table S4). Both HR and OR datasets were rated as very low certainty due to prominent confounding risks and other methodological limitations.

## 4. Discussion

Our updated meta-analysis included 18 full-text observational studies enrolling 8104 cirrhotic patients, and separately synthesized two distinct effect metrics (HRs/ORs). Clear divergent findings emerged between the two datasets, which substantially shapes our interpretation of the NSBB–PVT association and addresses major limitations of the prior meta-analysis published by Xu et al. (2019) [19]. Consistent with previous meta-analyses, crude pooled data supported a positive correlation between NSBB administration and elevated PVT risk. However, our stratified analyses challenge the reliability of this crude association and indicate that the independent relationship between NSBB exposure and PVT remains to be further verified. Importantly, the significant correlation disappeared after multivariable adjustment for multiple clinical confounders in longitudinal cohorts targeting de novo PVT, highlighting that the unadjusted positive signal may largely be driven by baseline imbalances rather than a direct thrombotic effect of NSBBs. The earlier review mixed abstracts and low-quality incomplete manuscripts, pooled heterogeneous OR/HR data together, and reported an exaggerated pooled OR of 4.62. In contrast, we strictly excluded conference abstracts, separated time-to-event HR analyses from binary OR analyses, and obtained a more moderate pooled OR of 1.89 (95% CI 1.29–2.78) for PVT risk, alongside a non-significant pooled HR of 1.61 (95% CI 0.99–2.63) for de novo thrombosis during follow-up. Given the strong potential for confounding by indication in observational research, adjusted effect estimates are given primary interpretive weight throughout this analysis.

Subgroup analyses explored sources of moderate-to-high heterogeneity (I^2^ = 67.8%) across the 12 OR studies. Adjustment status was a major contributor to inter-study variation: the subset of fully adjusted studies showed a significant positive association with no statistically detectable heterogeneity (OR = 2.47, 95% CI 1.36–4.50, I^2^ = 0%, *p* = 0.385), while crude unadjusted analyses also showed elevated PVT odds with substantial residual heterogeneity (OR = 1.74, 95% CI 1.12–2.69, I^2^ = 72.5%). Stratification by PVT phenotype yielded consistent positive associations in both de novo incident (3 studies, OR = 2.79) and non-incident PVT subgroups (9 studies, OR = 1.63). However, all OR-based signals are prone to confounding by indication and lack the time-to-event precision of Cox-derived HRs, limiting causal inference. Further stratification revealed inconsistent results by study design and region. Cohort studies reporting binary PVT outcomes showed a significant association (OR = 2.19, I^2^ = 36%), whereas case–control studies yielded a non-significant pooled estimate (OR = 1.68, 95% CI 0.94–3.01). Geographically, the association was significant in European cohorts (OR = 2.19) but not in Asian cohorts (OR = 1.41, 95% CI 0.64–3.11, I^2^ = 73.2%), highlighting regional variability that weakens support for the prothrombotic effect of NSBBs.

For the six longitudinal HR cohort studies exclusively assessing incident PVT, subgroup analysis limited to adjustment status yielded divergent trends. Crude unadjusted HRs produced a significant pooled risk estimate (HR = 1.96, 95% CI 1.04–3.71), yet multivariable-adjusted HRs attenuated to a null association (HR = 1.16, 95% CI 0.52–2.55). This marked attenuation after adjustment highlights that baseline clinical imbalance largely accounts for the crude positive signal, and current adjusted longitudinal data do not provide robust evidence that NSBBs independently increase incident PVT risk.

The previous theory of the prothrombotic effect of NSBBs is grounded in the hemodynamic effects of NSBBs and Virchow’s triad for thrombosis [12,16]. NSBBs reduce cardiac output by blocking β_1_-receptors and induce splanchnic vasoconstriction via β_2_-receptor antagonism [14,15], both of which may slow portal venous flow velocity [32]. In the setting of preexisting endothelial dysfunction, imbalanced procoagulant and anticoagulant factors, and chronic inflammation characteristic of end-stage liver disease, NSBB-mediated flow stasis may create a prothrombotic microenvironment in the portal vein [12,13]. However, clinical data from adjusted longitudinal cohorts failed to corroborate this hypothetical risk in real-world cirrhotic populations. Cirrhosis patients exhibit rebalanced coagulation function, and flow reduction induced by standard-dose NSBBs may not be sufficient to independently trigger de novo thrombosis after adjusting for all baseline thrombotic risk factors.

Evidence-based data from hepatic venous pressure gradient (HVPG) monitoring has established NSBBs as the cornerstone pharmacotherapy for the primary and secondary prophylaxis of variceal bleeding in patients with cirrhosis and clinically significant portal hypertension (defined as an HVPG ≥ 10 mmHg) [33,34]. Among all the NSBBs, carvedilol has been demonstrated in multiple studies to exert a more potent portal hypotensive effect than conventional NSBBs, owing to its unique pharmacological property of α1-adrenergic receptor blockade that induces splanchnic vasodilation [33,34]. A systematic review showed that carvedilol reduces HVPG by a mean of approximately 20% to 25%, whereas propranolol only achieves a 10% to 15% reduction [33]. However, most included studies did not separately report PVT risk by NSBB subtype, and data on dosage, treatment duration, and adherence was very limited. Given the distinct hemodynamic profiles of propranolol and carvedilol, treating all NSBBs as a single class is an important limitation of the current evidence base, and the available data are insufficient to support reliable subgroup analyses by individual agent.

Several notable limitations of the present meta-analysis warrant acknowledgement, which together moderate conclusions regarding the NSBB–PVT relationship. First, confounding by indication universally affects all included observational studies: patients receiving NSBB treatment typically present with advanced liver disease, severe portal hypertension and elevated baseline thrombotic risk, which readily explains crude positive correlations without attributing harm to NSBB therapy itself. Second, residual confounding remains an important concern: included studies adjusted for different combinations of clinical covariates, resulting in variable degrees of confounding control across studies. This heterogeneity in adjustment strategies limits direct comparability of effect sizes and means that residual unmeasured confounding cannot be excluded. Third, longitudinal HR evidence specifically targeting incident PVT has notable limitations beyond small sample size. On one hand, leave-one-out sensitivity analysis demonstrated that weak crude positive trends are unstable and highly sensitive to individual influential studies. On the other hand, the available adjusted effect sizes are incomplete. Among the three studies providing only crude unadjusted HRs, two [5,28] explicitly documented that the NSBB–PVT association was attenuated to non-significance after multivariable adjustment; yet full adjusted HR values with corresponding 95% CIs were not published, and our data requests to corresponding authors went unanswered. If these unreported null-adjusted estimates could be included in the pooled analysis, the neutral longitudinal association between NSBB therapy and de novo PVT would likely be further strengthened. Fourth, formal publication bias assessment has limited power given the small number of included studies; while Egger’s test did not reach statistical significance for the OR dataset, mild funnel plot asymmetry means that publication bias cannot be completely excluded. Fifth, follow-up durations varied markedly across cohorts; we emphasize that time-adjusted HR analyses are more credible for assessing de novo PVT risk under unequal observation windows. Sixth, diagnostic definitions and imaging criteria for PVT varied across primary studies, as fully documented in Supplementary Table S1. While all studies excluded malignant thrombus, imaging thresholds and evaluated venous segments varied. Meanwhile, most publications did not report PVT location or severity stratified by NSBB exposure, which may contribute to inter-study heterogeneity. Finally, the small sample size of Asian studies restricts global generalizability, though pooling results from this subgroup align with the tentative conclusion that NSBBs may not confer consistent thrombotic risk.

In conclusion, current observational evidence is insufficient to definitively confirm or refute an independent prothrombotic effect of NSBBs on de novo portal vein thrombosis in cirrhosis. While pooled OR analyses show a correlation between NSBB use and PVT, high-quality adjusted longitudinal HR cohort data fail to confirm that NSBB therapy independently raises the risk of incident PVT. Crude positive signals observed in unadjusted datasets are predominantly attributable to baseline clinical imbalance and confounding by indication.

From a clinical practice standpoint, current evidence does not justify routine discontinuation or avoidance of NSBBs in cirrhotic patients with clinically significant portal hypertension, given their well-established survival benefit in the primary and secondary prophylaxis of variceal hemorrhage. However, clinicians should be mindful of the potential thrombotic signal in patients with multiple concomitant prothrombotic risk factors (e.g., prior PVT, advanced cirrhosis, inherited hypercoagulable states). Large, long-term multinational prospective cohorts with comprehensive multivariable adjustment and uniform incident PVT endpoints are still needed to further validate the relationship.

## Figures and Tables

**Table 1:** Baseline characteristics of included studies.

First Author (Year)	Country	Study Design	Follow-Up Time	No. Patients	Pooled Estimates (HR) (95% Confidence Interval)	Adjusted Covariates	NOS Score
Simone (2025) [22]	Italy	Prospective Cohort	6 months	488	aHR 1.01 (0.52, 1.94)	Child–Pugh score, MELD Score	9
Fanny (2021) [3]	Spain	Prospective Cohort	>6 months	369	aHR 0.74 (0.15, 3.6)	Variceal bleeding, large esophageal varices	8
Filipe (2019) [4]	France	Prospective Cohort	19 months	108	aHR 6.15 (0.63, 59.96)	Esophageal varices	8
Carlos (2019) [28]	Portugal	Prospective Cohort	29 months	233	uHR 4.63 (1.64, 13.05)	Crude estimate	7
Andrea (2023) [20]	Italy	Prospective Cohort	5 years	202	uHR 1.24 (0.5, 3.1)	Crude estimate	7
Filipe (2015) [5]	France	Prospective Cohort	47 months	1243	uHR 1.67 (1.02, 2.73)	Crude estimate	8
Xiangbo (2020) [21]	China	Retrospective Cohort	>6 months	43	aOR 4.08 (0.49, 34.16)	Age, serum creatinine, Child–Pugh score	7
Marta (2021) [24]	Spain	Prospective Cohort	12 months	567	aOR 4.3 (1.4, 12.6)	Previous decompensation state, basal laboratory tests	8
Rosa (2018) [11]	Italy	Retrospective Case–Control	3 months	130	aOR 1.78 (1.03, 4.7)	Esophageal varices	6
Ellen (2021) [26]	Netherlands	Retrospective Case–Control	NA	78	uOR 5.5 (1.56, 19.36)	Crude estimate	7
Francesco (2016) [30]	Italy	Prospective Cohort	NA	753	uOR 1.43 (0.98, 2.11)	Crude estimate	6
Lydia (2018) [31]	Italy	Retrospective Case–Control	5 years	230	uOR 2.25 (0.87, 5.83)	Crude estimate	8
Monica (2022) [27]	Italy	Prospective Cohort	26 months	79	uOR 2.94 (0.46, 18.75)	Crude estimate	7
Ollivier (2022) [25]	France	Prospective Cohort	65.4 months	1789	uOR 2.54 (1.55, 4.18)	Crude estimate	8
Wen (2015) [29]	China	Retrospective Case–Control	NA	151	uOR 7.3 (2.33, 22.85)	Crude estimate	6
Xiaoquan (2020) [9]	China	Retrospective Case–control	12 months	124	uOR 0.84 (0.32, 2.2)	Crude estimate	8
Xiaoze (2025) [23]	China	Retrospective Case–control	NA	1389	uOR 1.08 (0.84, 1.4)	Crude estimate	8
Wenting (2025) [6]	China	Retrospective Case–control	NA	128	uOR 0.47 (0.17, 1.33)	Crude estimate	7

OR: odds ratio; HR: hazard ratio; aHR: adjusted HR; uHR: unadjusted HR; aOR: adjusted OR; uOR: unadjusted OR; NA: not available.

**Table 2:** Subgroup analyses for OR dataset.

Subgroup Analysis	Included Studies	Pooled OR (95% CI)	Heterogeneity I^2^ (%)	*p*
Adjustment status				
Adjusted estimates	3	2.47 (1.36, 4.50)	0.0%	0.385
Unadjusted estimates	9	1.74 (1.12, 2.69)	72.5%	<0.001
Overall	12	1.89 (1.29, 2.78)	67.8%	<0.001
Study design				
Cohort	5	2.19 (1.40, 3.42)	36.0%	0.182
Case–control	7	1.68 (0.94, 3.01)	73.0%	0.001
Overall	12	1.89 (1.29, 2.78)	67.8%	<0.001
PVT outcome type				
Incident PVT	3	2.79 (1.80, 4.32)	0.0%	0.692
Non-incident PVT	9	1.63 (1.07, 2.50)	66.2%	0.003
Overall	12	1.89 (1.29, 2.78)	67.8%	<0.001
Geographic region				
European	7	2.19 (1.55, 3.09)	29.3%	0.204
Asian	5	1.41 (0.64, 3.11)	73.2%	0.005
Overall	12	1.89 (1.29, 2.78)	67.8%	<0.001
Study quality				
NOS < 7	3	2.27 (1.03, 4.97)	71.7%	0.029
NOS ≥ 7	9	1.81 (1.09, 3.00)	69.2%	0.001
Overall	12	1.89 (1.29, 2.78)	67.8%	<0.001

OR: odds ratio; CI: confidence interval.

## Data Availability

All raw data were abstracted from previously published original studies. Readers may contact the corresponding author to acquire the analytical dataset and Stata command files for this meta-analysis with legitimate requests.

## Supplementary Materials

The following supporting information can be downloaded at: https://www.mdpi.com/article/10.3390/biomedicines14092034/s1, Figure S1: Subgroup analysis stratified by adjustment status. 1: adjusted estimates; 2: unadjusted estimates; Figure S2: Subgroup analysis stratified by study design. 1: cohort study; 2: case–control study; Figure S3: Subgroup analysis stratified by PVT outcome type. 1: incident PVT; 2: non-incident PVT; Figure S4: Subgroup analysis stratified by geographic region. 1: European region; 2: Asian region; Figure S5: Subgroup analysis stratified by study methodological quality. 1: NOS score < 7; 2: NOS score ≥ 7. Table S1: PVT definitions and diagnostic methods in eligible studies. Table S2: Quality assessment for cohort studies based on the Newcastle–Ottawa Scale; Table S3: Quality assessment for case–control studies based on the Newcastle–Ottawa Scale; Table S4: GRADE certainty assessment for the association between NSBB use and portal vein thrombosis risk; Table S5: NSBB-related information from included studies; Supplementary File S1: PRISMA 2020 Checklist [35]; Supplementary File S2: Full database-specific search strategies.

## Abbreviations

The following abbreviations are used in this manuscript:

NSBBs	Non-selective β-blockers
PVT	Portal vein thrombosis
NOS	Newcastle–Ottawa Scale
HVPG	Hepatic venous pressure gradient
