# Supplementary material for: Non-Selective β-Blocker Use and Portal Vein Thrombosis in Cirrhosis: An Updated Systematic Review and Meta-Analysis
Source: Biomedicines. 2026 Sep 10;14(9):2034. doi: 10.3390/biomedicines14092034 (PMC13604597; doi:10.3390/biomedicines14092034)
Supplement: Supplementary file 1 [file biomedicines-14-02034-s001.zip › Supplementary File S2.pdf]

Supplementary File S2. Full database-specific search strategies

Database	Electronic Search
**Medline/PubMed**	("Liver Cirrhosis"[Mesh] OR cirrhosis OR cirrhotic OR "hepatic cirrhosis") AND ("Portal Vein Thrombosis"[Mesh] OR "portal vein thrombosis" OR PVT OR portal thrombosis OR "portal venous thrombosis") AND ("Adrenergic beta-Antagonists"[Mesh] OR "non-selective beta blocker*" OR "nonselective beta blocker*" OR NSBB* OR propranolol OR carvedilol OR nadolol)
**Web of Science**	Title/Abstract/Keyword ("liver cirrhosis" OR cirrhosis OR cirrhotic OR "hepatic cirrhosis") AND Title/Abstract/Keywords ("portal vein thrombosis" OR PVT OR portal thrombosis OR "portal venous thrombosis" OR "splanchnic vein thrombosis") AND Title/Abstract/Keywords ("non-selective beta blocker*" OR "nonselective beta blocker*" OR NSBB* OR propranolol OR carvedilol OR nadolol)
**Cochrane Library**	Title/Abstract/Keyword ("liver cirrhosis" OR cirrhosis OR cirrhotic OR "hepatic cirrhosis") AND Title/Abstract/Keyword ("portal vein thrombosis" OR PVT OR portal thrombosis OR "portal venous thrombosis") AND Title/Abstract/Keyword ("non-selective beta blocker*" OR "nonselective beta blocker*" OR NSBB* OR propranolol OR carvedilol OR nadolol)
**EMBASE**	('liver cirrhosis'/exp OR cirrhosis: ti,ab OR cirrhotic: ti,ab OR "liver cirrhosis: ti,ab " OR "hepatic cirrhosis: ti,ab ") AND ('portal vein thrombosis'/exp OR "portal vein thrombosis: ti,ab " OR PVT: ti,ab OR portal thrombosis: ti,ab OR "portal venous thrombosis: ti,ab ") AND ('beta adrenoceptor antagonist'/exp OR "non-selective beta blocker*: ti,ab " OR "nonselective beta blocker*: ti,ab " OR NSBB*: ti,ab OR propranolol: ti,ab OR carvedilol: ti,ab OR nadolol: ti,ab)
