# Supplementary material for: Non-Selective β-Blocker Use and Portal Vein Thrombosis in Cirrhosis: An Updated Systematic Review and Meta-Analysis
Source: Biomedicines. 2026 Sep 10;14(9):2034. doi: 10.3390/biomedicines14092034 (PMC13604597; doi:10.3390/biomedicines14092034)
Supplement: Supplementary file 1 [file biomedicines-14-02034-s001.zip › Table S1.pdf]

Table S1. PVT definitions and diagnostic methods in eligible studies.

Study	PVT definition	Diagnostic methods	PVT outcome type
Simone (2025)	Clear thrombosis in the portal vein system	Doppler ultrasound, angio-CT, and/or angio-MRI	Incident PVT
Fanny (2021)	Presence of endoluminal material compatible with non-tumoral thrombosis in the portal vein and/or its branches.	CT or MRI	Incident PVT
Filipe (2019)	Solid hyperechoic material and a filling defect on Doppler study found within the main trunk of the portal vein and/or its branches	Doppler ultrasound	Incident PVT
Carlos (2019)	Solid endoluminal material detected in the main trunk of the portal vein and/or its branches with/without extension into the splenic or SMV	Doppler ultrasound	Incident PVT
Andrea (2023)	Endoluminal material found in the main portal trunk and/or in its extrahepatic (mesenteric and splenic vein) or intrahepatic branches	US Doppler	Incident PVT
Filipe (2015)	Solid endoluminal material detected in the main trunk of the portal vein and/or its branches	US Doppler	Incident PVT
Xiangbo (2020)	Thrombosis in any vessel of the portal venous system	Contrast-enhanced CT and/or MRI scans	Non-incident PVT
Marta (2021)	Hyperechoic material within the portal vein and absence of flow in some or all of the vessel lumen	Doppler ultrasound, angio-CT, and/or angio-MRI	Incident PVT
Rosa (2018)	Presence of a complete or partial obstruction of the main branch or afferent branches	Doppler ultrasound	Non-incident PVT

Ellen (2021)	Laminar appearance and the presence of chords or calcification	CT	Non-incident PVT
Francesco (2016)	Solid endoluminal material detected in the main trunk of the portal vein and/or its branches	Doppler ultrasound	Non-incident PVT
Lydia (2018)	Presence of intraluminal material in the portal vein or one of its branches	Doppler ultrasound, CT, and/or MRI	Non-incident PVT
Monica (2022)	Presence of endoluminal material compatible with non-tumoral thrombosis in the portal system (portal vein and/or its intrahepatic branches, splenic vein, superior mesenteric vein)	Doppler ultrasound, contrast enhanced CT	Incident PVT
Ollivier (2022)	Not specifically mentioned	Doppler ultrasound, contrast enhanced CT	Incident PVT
Wen (2015)	Complete or partial obstruction of the portal trunk, portal branches in the liver, and superior mesenteric vein	Doppler ultrasound, CT	Non-incident PVT
Xiaoquan (2020)	Not specifically mentioned	Doppler ultrasound, angio-CT	Non-incident PVT
Xiaoze (2025)	Not specifically mentioned	Doppler ultrasound, contrast enhanced CT or MRI	Non-incident PVT
Wenting (2025)	Existence of endoluminal material consistent with nontumoral thrombosis in the portal vein and/or its branches	Doppler ultrasound	Non-incident PVT

CT: Computed tomography; MRI: Magnetic resonance imaging
