# Supplementary material for: Non-Selective β-Blocker Use and Portal Vein Thrombosis in Cirrhosis: An Updated Systematic Review and Meta-Analysis
Source: Biomedicines. 2026 Sep 10;14(9):2034. doi: 10.3390/biomedicines14092034 (PMC13604597; doi:10.3390/biomedicines14092034)
Supplement: Supplementary file 1 [file biomedicines-14-02034-s001.zip › Table S2 .pdf]

Table S2. Quality assessment for cohort studies based on the Newcastle-Ottawa Scale.

Study	Selection				Comparability	Outcome			Score
	Representativeness of the exposed cohort	Selection of the non exposed cohort	Ascertainment of exposure	Demonstration that outcome of interest was not present at start of study	Comparability of cohorts on the basis of the design or analysis	Assessment of outcome	Was follow-up long enough for outcomes to occur?	Adequacy of follow-up of cohorts	
Andrea, 2023	*	*	*	*	*	-	*	*	7/9
Xiangbo, 2020	*	*	*	*	*	-	*	*	7/9
Simone, 2025	*	*	*	*	**	*	-	-	9/9
Marta, 2021	*	*	*	*	*	*	*	*	8/9
Ollivier, 2022	*	*	*	*	*	*	*	*	8/9
Monica, 2022	*	*	*	*	*	-	*	*	7/9
Fillipe, 2019	*	*	*	*	*	*	*	*	8/9
Fillipe, 2015	*	*	*	*	*	*	*	*	8/9
Francesco, 2016	*	*	*	*	*	*	-	-	6/9
Carlos, 2019	*	*	*	*	*	*	*	*	8/9
Fanny, 2021	*	*	*	*	**	*	*	*	9/9
