# Supplementary material for: Non-Selective β-Blocker Use and Portal Vein Thrombosis in Cirrhosis: An Updated Systematic Review and Meta-Analysis
Source: Biomedicines. 2026 Sep 10;14(9):2034. doi: 10.3390/biomedicines14092034 (PMC13604597; doi:10.3390/biomedicines14092034)
Supplement: Supplementary file 1 [file biomedicines-14-02034-s001.zip › Table S3.pdf]

Table S3. Quality assessment for case control studies based on the Newcastle-Ottawa Scale.

Study	Selection				Comparability	Exposure			Score
	Is the case definition adequate?	Representativeness of the cases	Selection of Controls	Definition of Controls	Comparability of cases and controls on the basis of the design or analysis	Ascertainment of exposure	Same method of ascertainment for cases and controls	Non-response rate	
Xiaoquan, 2020	*	*	*	*	**	*	*	-	8/9
Wenting, 2025	*	*	*	*	*	*	*	-	7/9
Xiaoze, 2025	*	*	*	*	**	*	*	-	8/9
Ellen, 2021	*	*	*	*	*	*	*	-	7/9
Wen, 2015	*	*	*	-	*	*	*	-	6/9
Rosa, 2018	*	-	*	*	*	*	*	-	6/9
Lydia, 2018	*	*	*	*	**	*	*	-	8/9
