# Supplementary material for: Non-Selective β-Blocker Use and Portal Vein Thrombosis in Cirrhosis: An Updated Systematic Review and Meta-Analysis
Source: Biomedicines. 2026 Sep 10;14(9):2034. doi: 10.3390/biomedicines14092034 (PMC13604597; doi:10.3390/biomedicines14092034)
Supplement: Supplementary file 1 [file biomedicines-14-02034-s001.zip › Table S4.pdf]

Table S4. GRADE certainty assessment for the association between NSBBs use and portal vein thrombosis risk

Certainty assessment						
**Studies**	**Risk of bias**	**Inconsistency**	**Indirectness**	**Imprecision**	**Publication bias**	**Overall certainty of evidence**
**Pooled HRs for PVT risk among NSBBs users**						
6 non - randomized cohort studies	serious	not serious	not serious	not serious	undetected	⊕◯◯◯ Very low
**Pooled ORs for PVT risk among NSBBs user**						
5 non-randomized cohort and 7 cases-control studies	serious	serious	serious	not serious	undetected	⊕◯◯◯ Very low

CI: confidence interval
