# Supplementary material for: Non-Selective β-Blocker Use and Portal Vein Thrombosis in Cirrhosis: An Updated Systematic Review and Meta-Analysis
Source: Biomedicines. 2026 Sep 10;14(9):2034. doi: 10.3390/biomedicines14092034 (PMC13604597; doi:10.3390/biomedicines14092034)
Supplement: Supplementary file 1 [file biomedicines-14-02034-s001.zip › Table S5.pdf]

Table S5. NSBB-related information from included studies.

Study	NSBB type	NSBB dose	Treatment duration
Simone (2025)	NA	NA	NA
Fanny (2021)	NA	NA	NA
Filipe (2019)	Carvedilol and propranolol	25mg Carvedilol/ 80mg Propranolol	NA
Carlos (2019)	Carvedilol and propranolol	NA	NA
Andrea (2023)	NA	NA	NA
Filipe (2015)	NA	NA	NA
Xiangbo (2020)	Propranolol	20mg	1.56 year
Marta (2021)	NA	NA	NA
Rosa (2018)	Propranolol	20mg	NA
Ellen (2021)	NA	NA	NA
Francesco (2016)	NA	NA	NA

Lydia (2018)	Carvedilol and propranolol	NA	24 months
Monica (2022)	NA	NA	NA
Ollivier (2022)	NA	NA	NA
Wen (2015)	NA	NA	NA
Xiaoquan (2020)	NA	NA	NA
Xiaoze (2025)	NA	NA	NA
Wenting (2025)	NA	NA	NA
